# An Integrated Outlook on the Metagenome and Metabolome of Intestinal Diseases

**DOI:** 10.3390/diseases3040341

**Published:** 2015-11-06

**Authors:** Wanping Aw, Shinji Fukuda

**Affiliations:** 1 Institute for Advanced Biosciences, Keio University, 246-2 Mizukami, Kakuganji, Tsuruoka, Yamagata 997-0052, Japan; E-Mail: wanping@sfc.keio.ac.jp; 2 RIKEN Center for Integrative Medical Sciences, 1-7-22 Suehiro-cho, Tsurumi-ku, Yokohama, Kanagawa 230-0045, Japan

**Keywords:** gut microbiota, luminal metabolites, colorectal cancer, inflammatory bowel disease, metabolomics, metagenomics

## Abstract

Recently, metagenomics and metabolomics are the two most rapidly advancing “omics” technologies. Metagenomics seeks to characterize the composition of microbial communities, their operations, and their dynamically co-evolving relationships with the habitats they occupy, whereas metabolomics studies unique chemical endpoints (metabolites) that specific cellular processes leave behind. Remarkable progress in DNA sequencing and mass spectrometry technologies has enabled the comprehensive collection of information on the gut microbiome and its metabolome in order to assess the influence of the gut microbiota on host physiology on a whole-systems level. Our gut microbiota, which consists of prokaryotic cells together with its metabolites, creates a unique gut ecosystem together with the host eukaryotic cells. In this review, we will highlight the detailed relationships between gut microbiota and its metabolites on host health and the pathogenesis of various intestinal diseases such as inflammatory bowel disease and colorectal cancer. Therapeutic interventions such as probiotic and prebiotic administrations and fecal microbiota transplantations will also be discussed. We would like to promote this unique biology-wide approach of incorporating metagenome and metabolome information as we believe that this can help us understand the intricate interplay between gut microbiota and host metabolism to a greater extent. This novel integration of microbiome, metatranscriptome, and metabolome information will help us have an improved holistic understanding of the complex mammalian superorganism, thereby allowing us to gain new and unprecedented insights to providing exciting novel therapeutic approaches for optimal intestinal health.

## 1. The Gut Microbiota

The gut microbiota refers to all the microorganisms inhabiting the gastrointestinal tract. In mammals, the gut microbiota is dominated by *Actinobacteria*, *Bacteroidetes*, *Firmicutes*, and *Proteobacteria*, and these phyla have been reported to play an important role in shaping host metabolism and physiology [1]. The total amount of bacteria populating the gut amounts to about 100 trillion cells, which is approximately three times higher than the total number of cells in the human body [2]. Thus, the gut microbiota is often considered to be a functional and measurable organ consisting of prokaryotic cells with host eukaryotic cells merging together to create a unique gut ecosystem [3]. According to the dietary lifestyle and nutritional status of the host, gut microbiota communities vary in composition along the digestive tract and evolve within and between individuals over time [4]. It is only in recent years that we have started to understand the systemic influence of the gut microbiota on the whole host metabolic repertoire. In addition to the gut microbiota’s obvious role in digestion, it plays a part in not only maintaining optimal host health but it is also involved the etiopathogenesis of various metabolic diseases such as obesity [5,6,7], diabetes [1,8,9]; intestinal diseases such as inflammatory bowel diseases (IBD) [10], colorectal cancer (CRC) [11]; and extraintestinal diseases such as allergies [12], multiple sclerosis [13], chronic kidney disease [9], atherosclerosis [14,15], and autism [16].

## 2. What Is Metabolomics?

Technological breakthroughs have enabled the comprehensive evaluation of thousands of genes (genomics), transcripts (transcriptomics), proteins (proteomics), metabolites (metabolomics), and gut microbiota (metagenomics) with high-throughput techniques and analytical tools [17] simultaneously. The rapid advances in DNA sequencing and mass spectrometry (MS) technologies in recent years have enabled the extensive collection of data on the gut microbiome and metabolome to comprehensively evaluate the impact of the gut microbiota on human health [18]. Since a holistic understanding of the organ and systemic metabolism is vital in maintaining health and nutritional status [19], this has led to major advances in metagenome and metabolome technologies to allow us to better understand the role that the gut microbiota play in influencing overall host health status.

Nuclear magnetic resonance (NMR) and MS are the two most commonly used wide-range metabolomic analytical methods in the identification of disease biomarkers. By using these approaches, we can accurately identify and have a robust understanding of the metabolites produced by microbiota and host cells in fecal, blood, tissue, and urine samples [20]. These tools allow scientists to comprehend the extent of the impact of treatments on the host metabolic profile by the simultaneous analysis of the presence and quantity of thousands of metabolites.

## 3. Using Metabolomics to Understand the Gut Microbiota

Nowadays, the evaluation of the metabolome profile is commonly used in the direct comparison of gut microbiota metabolism and the eventual metabolic outcomes in the host. In a report investigating the systemic influence of administering probiotics or prebiotics or a combination of both in initially germ-free mice colonized with a combination of microbes representing that of a human infant [21], it was revealed that probiotic/prebiotic intervention significantly modified the relative composition of the gut microbiota community, resulting in systemic changes in the metabolic profiles of different tissues. In groups administered prebiotics, it was observed that there were increased proportions of *Bifidobacterium breve*, *Bifidobacterium longum*, and *Bacteroides distasonis*; decreased proportions of *Escherichia coli* and *Clostridium perfringens*; and modulated lipid metabolism from decreasing concentrations of glucose and hepatic triglycerides in the plasma [21]. In another report by Wikoff *et al.* (2009), the effects of gut microbiota on the host were evaluated between germ-free and conventionally raised mice via comparing plasma metabolome profiles. There were many metabolites that were detected only in conventionally raised mice, and not in germ-free mice. In addition, concentrations of more than a tenth of all metabolites differed by more than 50% when comparing the conventionally raised mice and germ-free mice [22].

## 4. Relationships among the Gut Ecosystem, Colorectal Cancer and Inflammatory Bowel Disease

**Figure 1:**
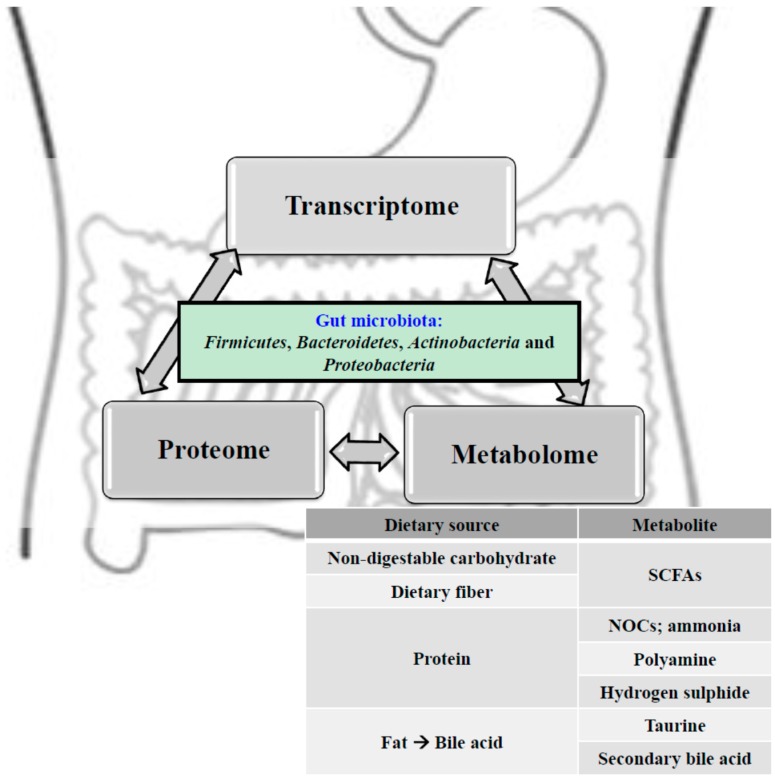
Our intricate gut ecosystem include four bacterial phyla: *Firmicutes*, *Bacteroidetes*, *Actinobacteria*, and *Proteobacteria* dominate the gut microbiota in mammals and these phyla have been reported to characterize the role of the host metabolism and physiology. Depending on the dietary lifestyle of the host, the gut microbiota and its metabolites such as *N*-nitroso compounds, ammonia, polyamine, taurine, bile acids, hydrogen sulphide, and short-chain fatty acids are highly implicated in the etiopathogenesis of metabolic diseases, intestinal diseases, and extraintestinal diseases, thereby playing a vital role in host health.

As we have reported previously, the integration between the activities of the gut microbiome and our genes reflects the overall human metabolism at the systemic level [23]. Our gastrointestinal tract provides nutrients to cells and tissues via the circulatory system, and likewise, so are the metabolites originating from the gut microbiota. This delicate interplay among gut microbiota-derived metabolites, the gut microbiota itself, and the host immune system is transmitted through an extensive array of signaling pathways that extend beyond the immune system. The direct chemical interactions between the gut microbiota and the host and the immune-mediated signaling mechanisms influence various organs such as the gut, liver, skeletal muscle, and brain, and these complex inter-relationships come together mutually to culminate in a series of host-microbe metabolic axes. Within these axes, metabolic reactions can be regulated by gut microbial genomes, resulting in the production of choline, phenols, bile acids, and short-chain fatty acids (SCFAs) by both the gut microbiome and host genome that are essential to host health [23] (Figure 1). In this review, we will discuss the relationships between the gut microbiota metabolism and protective and detrimental metabolites in the pathogenesis of CRC and IBD. In addition, we will also briefly discuss therapeutic interventions such as probiotic or prebiotic administration, and fecal microbiota transplantation (FMT).

## 5. Colorectal Cancer

CRC ranks third in cancer mortality causes worldwide. The mechanism behind CRC pathogenesis is known as the adenoma-carcinoma sequence [24] where genetic alterations result in the transition from the normal mucosa to pre-malignant lesions, then to colorectal adenomas and fulminant CRC over the years [25]. Although some causes of CRC are hereditary, most CRC cases can be attributed to diet and lifestyle [26]. However, a recent study has shown that diet-associated cancer progression is associated with significant shifts in gut microbial communities as a result of the host and environmental interaction independent of obesity, and that tumorigenesis may be transmissible among genetically predisposed individuals [27]. In addition, individuals with IBD have an increased incidence of CRC and this is known as colitis-associated cancer [28,29]. Inflammation itself has also been reported to alter host physiology, thereby promoting cancer, as seen in a murine model of colitis-associated CRC where colitis altered microbial composition and induced the increment of genotoxic microorganisms [30]. Although there are many reports that have reviewed the potential roles of particular strains of pathogenic bacteria in promoting CRC via pro-inflammatory interactions with host cells [25,29,31], it is progressively clear that the cumulative activities of the gut microbiota and their metabolic products significantly influence pathogenesis and protection against CRC [25,27,29,31,32].

## 6. Inflammatory Bowel Disease

IBD is a group of debilitating inflammatory disorders affecting the gastrointestinal tract. The two major types include Crohn’s disease (CD) and ulcerative colitis (UC). Neither of them is fatal; however, affected patients experience a large variety of symptoms associated with inflammation of the gut, ranging from abdominal pain, fever, vomiting, diarrhea, rectal bleeding, and anemia to weight loss. Symptoms are usually managed using anti-inflammatory steroids or immunosuppressants to reduce inflammation. Dietary or lifestyle interventions are also employed to try and remove environmental triggers. In severe cases of IBD, surgery is required to remove damaged portions of the bowel [33]. IBD prevalence is currently highest in Europe and North America [10]. IBD global prevalence is rising, with rapid increments in incidence rates occurring as more countries adopt a “Westernized” lifestyle [34]. Incidence rates are also rising in younger people, placing an increased strain on healthcare resources (particularly as early-onset IBD has been associated with a higher risk of developing CRC) [35]. A genome-wide association study in 2008 reported 30 significant susceptibility genes and loci for CD incidence and pathogenesis [36]. A 16S rRNA sequence evaluation of gastrointestinal content from CD and UC patients revealed an abnormal gut microbiota composition characterized by depletion of commensal *Firmicutes* and *Bacteroidetes* [37] and reduction of *Faecalibacterium prausnitzii*, a major member of the *Firmicutes* that has also been reported to be associated with CD [38]. Although there have been numerous reports about decreases in the diversity of gut microbial populations that are observed in CD and UC patients [23,37,38], a recent report showed that there are abnormal enteric viromes where a significant expansion of *Caudovirales* bacteriophages are observed in IBD patients. Interestingly, the viromes of CD and UC patients were disease- and cohort-specific and gut virome diversity was not secondary to changes in the gut microbial community. These data support a model in which changes in the virome may contribute to intestinal inflammation and bacterial dysbiosis, which allows for speculation about whether bacterial microbiome changes in IBD are secondary to changes in the emergence of bacteriophages or the introduction of bacteriophages from lifestyle interventions [39]. Overall, IBD represents a significant global health burden that is of growing concern.

## 7. Microbial Metabolism in the Gut

Typically, the anaerobic gut microbial community ferments undigested dietary components that reach the large intestine into a large range of metabolites. This illustrates both the amazing biochemical capacity of the gut microbiota as well as the large variety of available substrates [40]. The major fermentation products in healthy subjects include gases and organic acids, mainly three short-chain fatty acids (SCFAs): acetate, propionate, and butyrate. These SCFAs are often in a 3:1:1 ratio and have a combined concentration of 50–150 mM in the colon [41]. Non-digestible carbohydrates inclusive of non-starch polysaccharides (structural polysaccharides of plant cell walls), resistant starch, and certain soluble oligosaccharides (fructo-oligosaccharides) are usually the primary substrates for microbial fermentation [42].

Gut microbiota metabolism can also include anaerobic respiration where nitrate, sulphate, and several organic compounds function as electron acceptors [43]. Facultative anaerobes like Proteobacteria can use available oxygen as an electron acceptor which increases their energy recovery from substrates when compared to most obligate anaerobes, with the exception of *Bacteroides* spp. and *Faecalibacterium prausnitzii*. *Bacteroides* spp. have cytochromes while *F. prausnitzii* depend on extracellular electron transfer via flavins and thiols [44,45]. Gut microbes that utilize hydrogen and formate inclusive of acetogenic bacteria (e.g., *Blautia hydrogenotrophica*), methanogenic archaea (*Methanobrevibacter smithii*), and sulphate-reducing bacteria (e.g., *Desulfovibrio* spp.) have vital roles in anaerobic metabolism via interspecies cross-feeding interactions [46]. Relative contributions of acetogenesis, methanogenesis, and sulphate reduction by the methanogenic archaea are dependent on the abundance of these bacteria and variations in gut transit time [47].

## 8. Protective and Detrimental Metabolites

In this review, gut microbial metabolites and enzymatic activities would be divided into sections according to whether they are anticipated to be protective or have adverse effects on gut health, inflammation, and carcinogenesis.

## 9. Impact of SCFAs on Host Cells

SCFAs are rapidly absorbed from the intestinal lumen but their subsequent distribution, fate, and effects on host cell metabolism vary greatly. For instance, butyrate is utilized preferentially as an energy source by intestinal epithelial cells and thus has low concentrations in the systemic circulation. On the other hand, propionate is mostly metabolized in the liver and, as such, only acetate is present in relatively high concentrations ranging from 0.10 to 0.15 mM in peripheral blood [48]. Butyrate and propionate inhibit the activity of histone deacetylases (HDACs) in colonocytes and immune cells, thereby promoting the hyperacetylation of histones in addition to several transcription factors and proteins that are involved in signal transduction [49,50]. This influences gene expression and cellular differentiation in various ways, such as in the downregulation of pro-inflammatory cytokines interleukin (IL)-6 and IL-12 in colonic macrophages. SCFAs exert anti-inflammatory effects and have been reported to regulate colonic regulatory T cells (T_reg_ cells) in mice [51,52,53,54,55,56,57,58]. Recent reports show that the differentiation of T_reg_ cells that express transcription factor Foxp3, which is vital in controlling intestinal inflammation, are induced by butyrate and propionate [52,53,59]. Butyrate is presumed to cause elevated acetylation of histone H3 in the promoter and enhancer regions of the *Foxp3* locus, thereby leading to an increased expression of Foxp3 [53]. Propionate is assumed to function via the same mechanism, however, further investigation is required [51,52,53]. Although lactate has been reported to be involved in the inhibition of HDACs, the elevated concentrations required in the study may not be physiological [60].

Extracellular SCFAs are involved in several important interactions with surface-exposed receptors of host cells: G protein-coupled receptor 41 (GPR41), GPR43, and GPR109A [48,61,62]. GPR43 recognizes all three major SCFAs. The affinities for GPR41 are in the order propionate > butyrate >> acetate. On the other hand, GPR109A interacts only with butyrate [62]. Anti-inflammatory butyrate-driven signaling that involves GPR109A promotes the differentiation of T_reg_ cells and IL-10-producing T cells, blocking the activation of nuclear factor-kB (NF-Kb) as well as inducing apoptosis via a mechanism independent of HDAC inhibition [62,63]. Acetate and propionate bind to GPR43, thereby exerting anti-inflammatory effects via modulating T_reg_ cells [52,64]. As such, the cancer-protective effects of propionate and butyrate that are associated with high fiber intake may be mediated via the tumor-suppressing GPR43 and GPR109A genes [65]. Other reports of anti-carcinogenic effects of butyrate include inhibiting proliferation and selectively inducing apoptosis of CRC cells [49,51,66,67]. The cancer-suppressing effects have yet to be fully elucidated; however, HDAC inhibition may be the factor leading to changes in transcriptional regulation [50]. As a result of HDAC inhibition, butyrate, and to a smaller extent propionate, has been reported to activate the AP-1 signaling pathway in epithelial cell lines which play vital roles in controlling cell proliferation and apoptosis [68]. Despite the reported anti-cancer properties of butyrate, in recent years, a study using a CRC murine model demonstrated that low concentrations of butyrate might promote pathogenesis of CRC by stimulating the growth of colonic epithelial cells [27].

## 10. Detrimental Metabolites: Products of Protein Fermentation

Large amounts of protein intake result in an increase in the fermentation of diet-derived protein in the colon where elevated concentrations of amino acid-derived products such as branched-chain fatty acids and phenylacetic acid would be observed [69,70,71]. A subset of gut microbiota, including several substrains of *Bacteroides* spp. and some *Firmicutes*, ferment amino acids in order to produce potentially bioactive compounds such as phenylacetic acid, phenols, indoles, and *p*-cresol [72]. Several nitrogenous compounds, in particular *N*-nitroso compounds (NOCs), have the ability to promote cancer pathogenesis and exert carcinogenic effects via DNA alkylation, which may result in mutations. The intake of dietary NOCs is positively correlated with CRC incidence in Europeans [73]; however, NOCs can also be formed endogenously via both acid-driven nitrosation in the stomach and nitrosation of amines derived from the microbial fermentation of protein in the colon [74]. Elevated concentrations of fecal NOCs have been observed in individuals who consume high-protein diets in controlled dietary intervention studies [70]. Nitroreductases and nitrate reductases that are encoded by Proteobacteria are also a contributing factor towards nitrosation reactions. Ammonia, a product of protein fermentation, has been reported to be a potential carcinogenic agent, as relatively low concentrations have been reported to cause an increase in mucosal damage and colonic adenocarcinomas in murine models [71,74].

Polyamines like putrescine, spermidine, and spermine are involved in a wide range of essential physiological functions, such as in the maintenance of membrane structural integrity, and gene regulation and translation [75,76]. The major aforementioned polyamines are produced from arginine in host tissues but polyamine synthesis can also occur in the gut microbiota [77,78,79]. High levels of polyamines are reported to be toxic and associated with a plethora of diseases including cancer. Oxidative stress that results from polyamine catabolism has been attributed as the contributing factor towards toxicity [76]. Some gut bacteria such as *Bacteroides fragilis* upregulate polyamine production by host cells on top of contributing directly to the polyamine pool by synthesizing such compounds. Several pathogens, such as *Shigella flexneri*, *Streptococcus pneumonia*, *Salmonella enterica* subsp, and *Helicobacter pylori*, exploit polyamines to increase their virulence [75].

## 11. Detrimental Metabolites: Bile Acid Metabolism

The primary bile acids (or bile salts) cholic acid and chenodeoxycholic acid, which are synthesized in the human liver from cholesterol and are secreted in bile, mainly function to facilitate the metabolism of dietary fat and the absorption of fat-soluble vitamins and cholesterol. Primary bile acids undergo an enterohepatic cycle between the gut and the liver eight times per day, with 90%–95% of the bile acids being reabsorbed by the intestine and returned to the liver, whereupon they are conjugated to taurine in mice and to glycine in humans to form bile salts [80,81]. Approximately 5%–10% of the bile acids are biotransformed to a large extent through degradation by the gut microbiota, while some are lost in the feces. Gut microbiota involved in the biotransformation are mainly anaerobic, and belong to the genera *Bacteroides*, *Eubacterium*, and *Clostridium*. Taurine- and glycine-conjugated bile acids are deconjugated via bile acid hydrolases to their respective unconjugated free bile acids which then form secondary bile acids such as deoxycholic acid (DCA) and lithocholic acid [81,82] which are then reabsorbed, mainly by both bile acid transporters in the ileum and passive absorption in the intestine [82]. High-fat diets, which are reportedly positively correlated with CRC incidence, lead to increased bile acid secretion and increased fecal bile acid secretions that have been reported in CRC patients [83,84,85]. Bile acids are implicated in the carcinogenesis of different regions of the intestinal tract and associated tissues due to the generation of reactive oxygen species and reactive nitrogen species, of which both have been reported to cause DNA damage [84]. Animal experiments have reported that bile acid administrations have resulted in an increase in the number of tumors in the gut [84]. In a study where various concentrations of cholic acid-containing diets were fed to rats, it was observed that cholic acid modified the composition of gut microbiota in rats in a manner similar to that of high-fat diets [86]. As reported by David *et al.* in 2014, *Bilophila wadsworthia* growth is stimulated in mice by secreted bile acids while consuming saturated fats from milk and it is stimulated in humans who consume high-fat diets. Levels of *B. wadsworthia*, which contains the microbial genus *Bilophila*, increased, and this is correlated with long-term daily saturated fat intake. The animal-based diet also led to elevated fecal bile acid levels, and increases in the abundance of microbial DNA and RNA encoding sulphite reductases, leading to the conclusion that animal-based diets may induce changes to bile acid concentration and gut microbiota composition, thereby leading to the development of IBD [87].

## 12. Detrimental Metabolites: Hydrogen Sulphide

In the gut, hydrogen sulphide is produced via the reduction of diet-derived sulphate and the metabolism of compounds such as sulphur amino acids [85] and taurine [88]. Sulphate-reducing bacteria that are related to *Desulfovibrio* spp., although detectable in low numbers in most individuals, are able to use lactate as a co-substrate for growth and at the same time form sulphide [88]. Sulphide is toxic to colonocytes and inhibits butyrate oxidation, thereby resulting in the breakdown of the colonocyte barrier, promoting the pathogenesis of UC [89]. It can also damage DNA in non-transformed human cell lines at concentrations similarly detected in the colonic lumen (0.25–2 mM) [89] and reactive oxygen species are proposed to be involved in this genotoxicity [90].

## 13. Therapeutic Interventions of Intestinal Diseases

### 13.1. Probiotic Interventions

Probiotics are defined as “live microorganisms” and, when administered in appropriate amounts, exert beneficial effects on host health [91]. Specific bacterial strains have been reported to play a protective role against IBD by competing with pathogenic microbes or directly preventing colonization in the gut, as well as via their anti-inflammatory properties [92].

A non-pathogenic *E. coli*, *E. coli* Nissle 1917, is the best-studied single-strain probiotic. It has been reported to be as effective and safe as mesalazine in the maintenance of remission in UC patients [93]. In addition, the rectal administration of *E. coli* Nissle 1917 was reportedly significantly more effective than a placebo in inducing remission in distal mild-to-moderate active UC patients [94]. *Lactobacillus rhamnosus* GG was also compared against mesalazine for the maintenance of UC remission. Although similar relapse rates after six to 12 months of treatment were observed with both treatments, a significantly longer relapse-free time was obtained with *L. rhamnosus* GG [95]. *L. rhamnosus* GG was also used an adjunct interventional to conventional therapy for both the induction and remission maintenance of CD. However, no significant beneficial effects were observed over the placebo. In an animal study, when fermented milk containing *B. lactis*, *L. lactis*, *S. thermophilus*, and *L. bulgaricus* was administered to colitic mice, amelioration of inflammation was observed. There was also an increase in butyrate-producing bacteria, and a concomitant decrease of enterobacteriaceae strains *Klebsiella pneumoniae* and *Proteus mirabilis*, which were capable of inducing colonic inflammation [96]. When human baby microbiota-associated mice were treated with the probiotics *Lactobacillus paracasei* or *Lactobacillus rhamnosus* and two galactosyl-oligosaccharide prebiotics, the numbers of *Bifidobacterium longum* and *Bifidobacterium breve* were increased, whereas the numbers of *Clostridium perfringens* were lowered. This gut microbiota composition remodeling has resulted in changes in various host metabolic pathways such as gluconeogenesis, lipid profiles, and amino acid metabolism [97]. Conjugated linoleic acid is a naturally occurring isomer of linoleic acid found in ruminant-derived meat and dairy products [98] and has been reported to protect against colon carcinogenesis, atherosclerosis, and obesity in mice [99,100]. In a recent study where *Bifidobacterium longum* BB536 (BB536) was administered to gnotobiotic mice harboring 15 strains of predominant human gut-derived microbiota, it was observed that there was a significant increase in fecal levels of biotin precursor-pimelate, butyrate, and biotin in the BB536 group. The increase in biotin concentrations could be attributed to changes in metabolism related to biotin synthesis by *Bacteroides caccae* in mice. The proportion of butyrate-producing microbiota, *Eubacterium rectale*, was significantly higher in the BB536 group than in the group without [101].

### 13.2. Prebiotic Interventions

Prebiotics are non-digestible compounds that confer specific changes in the composition and activity of the gut microbiota, thereby exerting beneficial effects on host health [97]. Germinated barley foodstuff (GBF), which is frequently studied in IBD maintenance, is a prebiotic rich in glutamine and hemicellulose [102]. GBF dietary intervention resulted in an increase in SCFA production in murine models and decrease in bowel movements as well as amelioration of colon damage and fecal blood [102,103]. In small clinical trials with mild-to-moderate active UC patients, the administration of GBF as an adjunct to conventional therapy also presented with significant improvement of clinical activity scores. An increase in fecal butyrate was also reported in these subjects [104]. In addition, when used in dietary interventions in patients with quiescent UC for the maintenance of remission, GBF-intervened subjects had significantly lower recurrence rates than those with conventional therapy alone, accompanied with evident decreases in serum proinflammatory cytokines IL-6 and IL-8 [105]. In a recent placebo-controlled clinical trial with mild-to-moderate active or quiescent CD subjects, an oligofructose-enriched inulin intervention for four weeks was effective in reducing disease activity and modifying the gut microbiota composition by increasing the number of *B. longum* strains [106]. There are numerous animal model studies using prebiotic feed supplements that have shown a significant impact in the prevention and treatment of CRC. Feeding long-chain inulin-type fructans has increased bifidogenic effects, lowered pH, modulated immunity, and reduced the number of azoxymethane (AOM)-induced colonic pre-neoplastic aberrant crypt foci (ACF), and small intestinal and colon tumors in the CRC murine model [107]. Xylooligosaccharide and fructooligosaccharide intervention inhibited colonic ACF in dimethylhyrazine-treated rats by lowering cecal pH and serum triglyceride levels. This intervention also resulted in an increase in total cecal weight, an elevation in bifidobacterial population, and a signification reduction in colonic ACF [108].

### 13.3. Fecal Microbiota Transplantations

**Figure 2:**
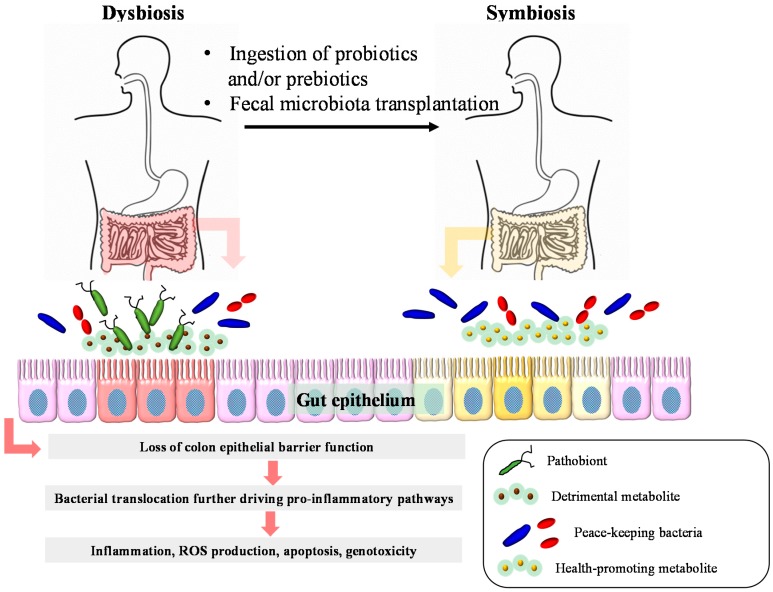
Dysbiosis-related inflammatory bowel diseases and colorectal cancer and relevant therapeutic interventions. Dysbiosis refers to the imbalance between the peace-keeping bacteria and the pathobionts, leading to intestinal diseases such as inflammatory bowel disease and colorectal cancer. Some bacterial metabolites can cause direct DNA damage or promote inflammation. Pathobionts also exert pro-inflammatory effects. In addition, the loss of barrier function will result in increased bacterial translocation, further driving pro-inflammatory pathways, resulting in tumorigenesis. Ingestion of probiotics and/or prebiotics and fecal microbiota transplantation have been reported to restore symbiosis.

In addition to the prebiotic and/or probiotic treatment, FMT can also potentially modulate the gut microbiota composition in order to improve the pathogenesis of various diseases such as chronic gastrointestinal infections, IBD, insulin resistance, multiple sclerosis, and idiopathic thrombocytopenic purpura [109]. FMT has been reported to be beneficial in antibiotic-associated diarrhea or *Clostridium difficile* infection [110,111,112]. FMT has also been reported to improve the quality of life of IBD patients [113]. IBD patients, including Ulcerative colitis (UC) and Crohn's disease (CD), were treated with FMT via colonoscopy or nasojejunal tube infusion and quality of life was documented by the subjects responding to an IBD questionnaire. Disease activity and the IBD questionnaire were evaluated at enrolment and four weeks after treatment. Patients’ attitudes concerning the treatment were also investigated. FMT improved the quality of life significantly in patients with IBD as observed in the significant decreases in the mean Mayo score in UC patients and the significant increases in the mean IBD questionnaire scores of both UC and CD patients four weeks after FMT treatment [113]. In another study, FMT from a single donor was administered via 22–30 treatments delivered by means of colonoscopy and enemas during a six- to 12-week period to three immunotherapy (infliximab, 6-mercaptopurine, and steroid, respectively)-dependent pediatric UC patients [114]. Patients were concomitantly withdrawn from their conventional medications. Mucosal disease activity was evaluated before and two weeks after the FMT treatment. Clinical disease activity and the Paediatric Ulcerative Colitis Activity Index (PUCAI) were also measured. FMT treatment was well tolerated and transiently supported immunotherapy withdrawal. FMT enabled all three patients to be symptom-free for at least four weeks following FMT and supported the withdrawal of immunotherapy. In addition, all subjects went into endoscopic and histologic remission two weeks after the last FMT [114]. Collectively, the improvement of the gut microbiota composition by fecal microbiota transplantation or treatment with probiotics and/or prebiotics may be beneficial in the prevention and medical treatment of several dysbiosis-associated disorders (Figure 2).

## 14. Conclusions

The studies that we have reviewed here highlight that that the pathogenesis of intestinal diseases such as IBD and CRC is not only dependent on specific pathogens, but also on the metabolic output by gut microbiota. However, we are still beginning to comprehensively understand the relationship between the protective and detrimental metabolites, their degradation pathway in the intestine by gut microbiota, and the subsequent impact on host health. Therefore, in light of this, we strongly advocate for the integration of metagenomic and metabolomic information as we believe that it is a valuable methodology that would enable us to further understand this intricate interplay between the gut microbiota and the host metabolic flux to a greater extent. On top of this, the integration of information derived from microbiome, metatranscriptome, and metabolome platforms will also lead to an improved comprehensive understanding of the complex mammalian superorganism. The vast amount of valuable data obtained from this multi-omics-based understanding of the metabolic interactions between lifestyle, nutritional interventions, and the gut ecosystem will provide intriguing novel therapeutic avenues not only in the prevention and maintenance of remission of CRC and IBD, but also in making vital contributions towards maintaining and promoting optimal host health for a higher quality of life for everyone.
